# Synthesis and Characterization of Novel Organotin-Phosphorous Compounds II

**DOI:** 10.3390/molecules15031425

**Published:** 2010-03-08

**Authors:** Salem S. Al-Deyab, Mohamed H. El-Newehy

**Affiliations:** Department of Chemistry, College of Science, King Saud University, Riyadh 11451, P.O. Box 2455, Saudi Arabia; E-Mail: ssdeyab@ksu.edu.sa (S.S.A.)

**Keywords:** organotin, phosphorous compounds, biocidal activity, antioxidants, organic synthesis intermediates

## Abstract

New organotin substituted α-anilinomethylphosphonates were prepared and were characterized by FT-IR, ^1^H- and ^13^C-NMR spectroscopy and elemental microanalysis.

## 1. Introduction

Many organometallic compounds exhibit interesting antitumour activity against several human cancer cell lines, and organotin(IV) compounds are a widely studied class of metal-based antitumour drugs [1]. The considerable interest in recent decades in the use of organotin compounds as reagents [2] or intermediates [3] in organic synthesis has led to the preparation of many new organotin compounds [4]. Organotins have been used in industrial and agricultural applications [5,6], as plastic stabilizers and catalysts, antifouling paints, molluscicides, fungicides [7] and disinfectants [8]. The introduction of biocidal organotin groups into phosphorous compounds could possibly enhance their biocidal activities [9]. The present study aimed to develop a new technique for the preparation of a new organotin substituted α-anilinomethylphosphonates.

## 2. Results and Discussion

**Figure 1:**
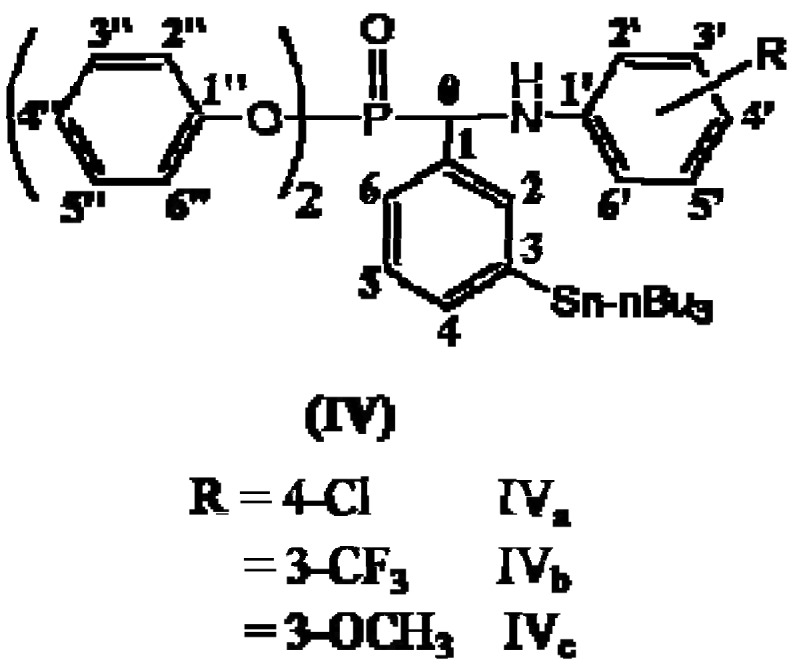
The structure of Compounds IV_a-c__._

A series of novel tri-n-butylstannyl phenyl substituted methylphosphonates IV_a-c_ (Figure 1) have been prepared *via* a Schiff base intermediate. 

**Scheme 1:**
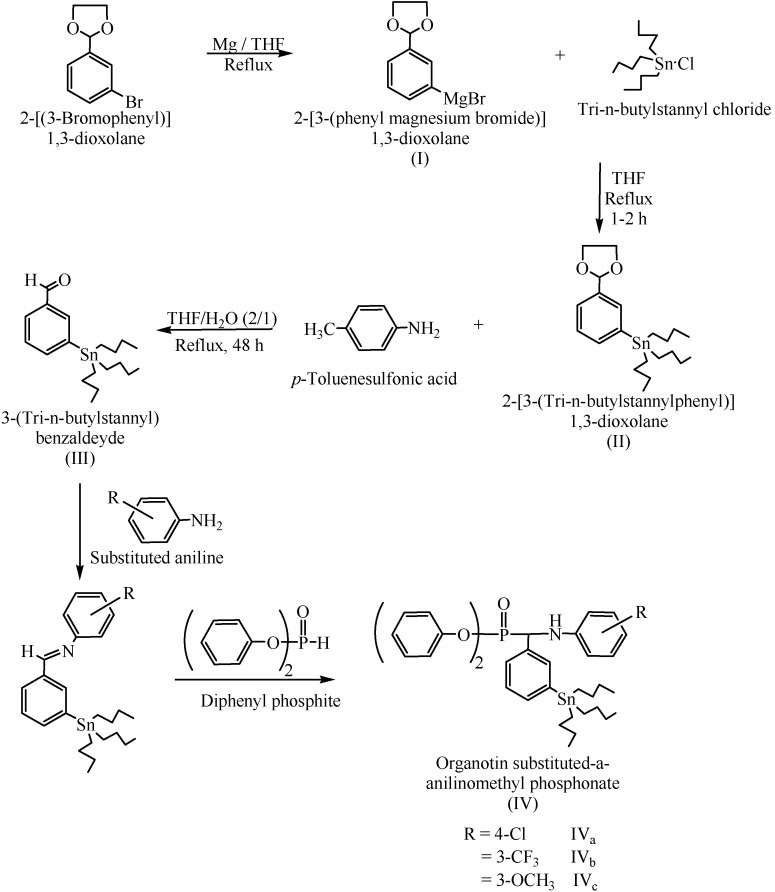
Synthesis of organotin-phosphorus compounds.

The active alkyltin groups were first attached to the *meta* position of the benzene ring [9,10] and the resulting aldehyde was then allowed to react with aniline and with a selected substituted aniline [9] such as *p*-chloroaniline, *m*-trifluoromethylaniline, and *m*-methoxyaniline giving a highly substituted Schiff base (imine) which is reacted directly with a phosphate ester such as diphenyl phosphate to give solid producs IV_a-c_ (Scheme 1).

The titled compounds were isolated in almost quantitative yield (Table 1). The stoichiometry of the compounds was established by elemental analysis (Table 2) which showed agreement with the calculated values; further evidence was obtained from the corresponding ^13^C-, ^1^H-NMR and FT-IR spectra.

**Table 1:** Physical properties of the title compounds.

Compound	R	Melting point (°C)	Yield (%)
IV_a_	4-Cl	81-82	87
IV_b_	3-CF_3_	87.5	58
IV_c_	3-OCH_3_	50-53	53

**Table 2:** Elemental microanalysis of the title compounds.

Compound	Calculated			Measured		
	%C	%H	%N	%C	%H	%N
II	57.43	8.26	-	57.63	8.33	-
III	61.09	8.72	-	61.18	8.64	-
IV_a_	60.14	6.41	1.90	60.02	6.37	1.93
IV_b_	59.09	6.13	1.81	58.79	6.21	1.87
IV_c_	62.14	6.86	1.91	62.21	6.93	1.94

### 2.1. ^13^C-NMR Spectra

Generally, the assignments of the ^13^C-NMR resonances for tin-phosphorous compounds (Table 3) in which there are more than three benzene rings are much more difficult than for the starting compounds (I, II, benzaldehyde, aniline, *p*-chloroaniline, *m*-trifluoromethylaniline, *m*-methoxyaniline, and diphenyl phosphite) (Table 4), because excluding the quaternary carbons, the ^13^C chemical shifts for the ring carbons in 1, 2 and 3 and its derivatives are spread over 18 ppm, compared with the ^13^C of the starting material in which only one benzene ring is found. Assignments of the ^13^C chemical shifts of rings 1, 2 and 3 were based on comparisons with reported values [9,11,12,13,14].

The quaternary carbons C-1, C-3, C'-1, C'-3, C'-4, C'-5, and C''-1 are readily identified since they are less intense and almost invariant in position compared with other signals, as a result of long relaxation times of the quaternary carbons [9,15].

For example, the ^13^C-NMR spectrum of diphenyl-1-(4-chloroanilino)-1-[3-(tri-*n*-butylstannyl)-phenyl]methyl phosphonate (**IV_a_**) in CDCl_3_, shows that the ^13^C signal of C_0_ (for the numbering refer to Figure 1) appears at δ 53.15 and 59.32 ppm, which indicates a clear spin-spin coupling between ^31^P and ^13^C.

The ring carbon values were confirmed by using the substituent chemical shift (SCS) effect of the ‑SnBu_3_^n^, -N-R, and (Ph-O)_2_P(O)-CH groups on the ring carbons, in comparison with the parent compounds. However, it has been found that the SCS effects for the above mentioned groups are additive in all positions. It is worth noting that the ^13^C chemical shift of C_0_ in which the phosphorous atom appears directly bonded to it generally appears as a doublet centered at δ 55.5 ppm. As expected the carbon of C_0_, appears as a single peak coupled with the adjacent ^31^P atom (*I* = 0.5, 100%).

^13^C spin-spin coupling constants involving ^31^P have been determined during the rapid growth of organophosphorous chemistry, particularly for biological molecules such as the nucleotides, phospholipids, and the titled compounds which contain phosphorous. ^13^C-^31^P spin-spin interactions have frequently been used as a probe and also used to identify carbons near the phosphorous atom. The ^13^C-NMR signal of the imine group (–C=N) of compound III (Scheme 1) shows only one signal at δ 161.0 ppm, which indicates the existence of only one isomer.

**Table 3:** ^13^C-NMR data of the titled compounds.

R group	δ (ppm)																		
	Sn-CH_2_-			-CH_2_CH_2_-			-CH_3_			C_0_		C_1_		C_2_		C_3_	C_4_	C_5_	C_6_
4-Cl	29.0			27.8			9.6			59.2		136.0		136.5		146.8	143.3	128.2	129.5
				13.6						53.0									
3-CF_3_	29.0			27.3			9.6			59.4		136.4		137.0		142.4	142.4	128.3	129.5
				13.6						53.2									
3-OCH_3_	29.0			27.3			9.6			60.1		134.3		136.3		142.4	142.4	127.9	128.3
				13.5						54.0									
**R group**	**δ (ppm)**																					
	**C'_1_**		**C'_2_**			**C'_3_**			**C'_4_**		**C'_5_**		**C'_6_**									
4-Cl	147.4		113.9			135.0			118.1		129.7		112.2									
3-CF_3_	146.4		115.1			139.0			119.3		129.0		120.8									
3-OCH_3_	142.6		91.9			153.8			120.4		153.8		91.9									
**R group**	**δ (ppm)**																			
	**C''_1_**	**C''_2,6_^a^**			**C''_3,5_^a^**			**C''_4_**												
4-Cl	150.4	120.7			130.2			127.3												
	149.8	120.5			130.2															
3-CF_3_	150.5	120.6			129.5			127.9												
	150.1	120.4			129.5															
3-OCH_3_	150.5	120.7			130.9			127.8												
	150.4	120.6			130.9															

^a ^two non-equivalent carbons.

**Table 4:** ^13^C NMR data of the starting compounds.

Compound	δ (ppm)													
	Sn-CH_2_-	-CH_2_CH_2_-		-CH_3_			C_0_		C_1_	C_2_	C_3_	C_4_	C_5_	C_6_
Sn-(CH_2_-CH_2_-CH_2_-CH_3_)_3_	27.8	26.8		13.6			-		-	-	-	-	-	-
		18.0												
III	29.1	27.4		9.7			192.2		135.6	137.7	143.6	142.6	128.4	129.5
		13.7												
**Compound**		**δ (ppm)**																			
		**C'_1_**	**C'_2_**		**C'_3_**			**C'_4_**		**C'_5_**	**C'_6_**										
4-Chloroaniline		147.7	114.8		134.7			118.2		130.3	113.2										
3-Trifluoromethaneaniline		146.4	115.8		138.9			119.3		129.7	112.2										
3-Methoxyaniline		143.3	98.8		152.9			119.0		152.9	98.8										
**Compound**		**δ (ppm)**																	
		**C''_1_**	**C''_2,6_^a^**			**C''_3,5_^a^**		**C''_4_**											
Diphenyl phosphite		149.3	120.8			131.8		127.6											
		149.0	120.5			131.8													

^a ^two non-equivalent carbons.

### 2.2. ^1^H-NMR Spectra

The assignments of the ^1^H Spectra for tin-phosphorous compounds were summarized in (Table 5).

**Table 5:** ^1^H-NMR data of the titled compounds.

Compound	δ (ppm)				
	3(-(C *H_2_)_3_*C*H_3_*)	*Ar-H*	*-O-CH-O-*	-O(C *H_2_*)*_2_*O-	-C *H*O
**II**	1.2 (*m*, 27H)	7.5 (*m*, 4H)	5.8 (*s,* 1H)	4.1 (*d,* 4H)	*-*
**III**	1.2 (*m*, 27H)	7.7 (*m*, 4H)	*-*	*-*	10.3 (*s,* 1H)
**Compound**	**δ (ppm)**				
	**3(-(C*H_2_)_3_*C*H_3_*)**	**-P-C*H*-N-**	**-N*H*-**	***Ar-H***	
**IV_a_**	0.6-1.8 (*m*, 27H)	4.7 (*m*, 1H)	5.4 (*d*, 1H)	6.5-6.7 (*m*, 18H)	
**IV_b_**	0.6-1.7 (*m*, 27H)	4.8-5.0 (*d*, 1H)	5.3-5.4 (*m*, 1H)	6.6-7.6 (*m*, 18H)	
**IV_c_**	0.6-1.6 (*m*, 27H)	3.8 (*s*, 1H)	5.4 (*d*, 1H)	6.25-7.6 (*s*, 18H)	

### 2.3. FT-IR Spectra

The structures of the titled compounds were further confirmed by their FT-IR spectra as shown in (Table 6).

**Table 6:** FT-IR data of the titled compounds.

Compound	Wavenumber (cm^-1^)							
	-(CH_2_)_3_CH_3_		Aromatic ring	P-O-Aryl	-P=O	C-O-C		-C=O
	**C-H stretching**	**C-H bending**	**C=C stretching**	**Stretching**				
**II**	2860, 2910, 2940	1350, 1370	1420, 1455	-	-	1080	-	
**III**	2840, 2910, 2950	1360	1450	-	-	-	1700	
**IV_a_**	2900, 2940	1290	1480	1180	1200	-	-	
**IV_b_**	2840, 2900, 2960	1340	1480	1100	1200	-	-	
**IV_c_**	2920, 2940	1300	1470	1040	1200	-	-	

Generally, the conversion of compound **II** into compound **III** was confirmed by the disappearance of the peak at 1080 cm^-1^ and the appearance of a peak at 1700 cm^-1^ which was assigned to C=O stretching. Moreover the formation of compounds **IV_a-c_** was confirmed by peaks at 760, 1150 and 1260 cm^-1^ which were assigned to C-Cl, C-F and -O-CH_3_ stretching, respectively. In addition, the formation of compounds **IV_a-c_** was confirmed *via* the FT-IR spectra by the disappearance of peaks at 1700 cm^-1^ and the appearance of peaks at 3290, 3310, 3320 cm^-1^ and at 1580 cm^-1^ assigned to N-H stretching and bending, respectively.

## 3. Experimental

### 3.1. Instruments

^1^H- and ^13^C-NMR spectra were recorded on a Jeol JNM FX-100 spectrometer operating in the Fourier Transform mode. All the spectra were recorded at ambient temperature. The compounds were dissolved in CDCl_3_ (concentration 50–100 mg in 2 mL solvent using a 10 mm diameter NMR tube). Chemical shift data were determined relative to the internal standard TMS. Melting points were determined using a Melt-temp melting point apparatus and are uncorrected, as were the boiling points. Elemental analysis were performed at M-H-W Laboratories (Phoenix, AZ, USA). FTIR spectra were recorded on Perkin Elmer 883 (Research Center, College of Science, King Saud University). Thin-layer chromatography (TLC) was performed using the ascending technique with silica gel 60F 254 precoated aluminium sheets.

### 3.2. Methods

Organotin substituted α-anilinomethyl phosphonates such as diphenyl-1-(4-chloroanilino)-1-[3-(tri-*n*-butylstannyl)-phenyl]methyl phosphonate (**IV_a_**) was prepared as follows:

#### 3.2.1. Preparation of 2-[3-(tri-n-butylstannyl)]1,3-dioxolane (**II**)

In a 500 mL three-neck round bottom flask equipped with two dropping funnels, a reflux condenser, and magnetic stirrer, a suspension of magnesium turnings (1.5 g, 60 mmol) in dry tetrahydrofuran (10 mL) was heated under gentle reflux. A solution of 2[(3-bromophenyl)] 1,3-dioxolane (8 g, 35 mmol) and 1,2-dibromoethane (4.5 g, 24 mmol) in dry tetrahydrofuran (100 mL) was prepared and 5 mL of this solution was added to the magnesium. Several drops of 1,2-dibromoethane were further added to initiate a vigorous reaction. The remaining dioxolane/1,2-dibromoethane solution was added dropwise under refluxing conditions, with occasional stirring, at the same time (tri-*n*-butylstannyl chloride (10 g, 30 mmol) in dry tetrahydrofuran (30 mL) was added. After the additions were completed, the remaining 1,2-dibromoethane was added in portions to destroy the remaining magnesium metal. After the reaction was completed (1–2 h) the mixture was stirred for 30 min at 50 °C and then allowed to cool with continuous stirring for 40 min. The mixture was hydrolyzed and washed with saturated ammonium chloride solution. The organic layer was separated and the aqueous layer was extracted twice with benzene (70 mL), and the combined organic layers were dried over MgSO_4_. The solvents were stripped off using a rotavapor and the remaining liquid was fractionally distilled twice under reduced pressure to give 9.93 g (73%) yield) of the desired compound **II** as a colorless liquid (b.p. 145–147 °C/0.2 mmHg).

#### 3.2.2. Preparation of 3-(tri-n-butylstannyl)-benzaldehyde (**III**)

3-(Tri-*n*-butylstannyl)-benzaldehyde was prepared by dissolving 2-[3-(tri-*n*-butylstannylphenyl)]- 1,3-dioxolane in THF (100 mL) and water (50 mL) containing *p*-toluenesulfonic acid (1 g). This solution was gentle refluxed under an inert atmosphere. After 48 h, the organic layer was separated and the aqueous layer was extracted twice with portions of benzene (50 mL), and the combined organic layers were dried over MgSO_4_. The solvents were stripped off and the remaining liquid was fractionally distilled under reduced pressure to give a yield of 89–93% of **III** as a colorless compound (b.p. 140–142 °C/0.07 mmHg).

#### 3.2.3. Preparation of organotin substituted α-anilinomethyl phosphonates IV

3-(tri-*n*-Butylstannyl)-benzaldehyde (**III**, 1 equiv.) was placed in a 25 mL conical flask and then one equivalent of substituted aniline was added. The mixture was gently heated on a hotplate, with occasional stirring. Upon slightly cooling an equivalent of diphenyl phosphite was added, and the resulting mixture was heated for a short time with continuous stirring, until the viscosity of the media increased. After a few mL of methanol were added, the solution was stored in a refrigerator for several hours until a precipitate was formed, which was filtered and recrystallized from methanol twice.

## 4. Conclusions

A series of novel tri-*n*-butylstannyl phenyl substituted methyl phosphonated were prepared in a good yield *via* Schiff bases and their structures were confirmed by FT-IR, ^1^H- and ^13^C-NMR spectroscopy and elemental microanalysis.
